# Sleep duration and cognitive function among rural older adults in China: A population-based study

**DOI:** 10.1371/journal.pone.0318044

**Published:** 2025-10-22

**Authors:** Yongxu Fang, Zhongrui Yan, Xinglu Wang, Rui She, Peng Wang, Yajun Liang

**Affiliations:** 1 College of Medicine, Jining Medical University, Jining, Shandong, China; 2 Department of Neurology, Jining No. 1 People’s Hospital, Jining, Shandong, China; 3 Department of Geriatric Medicine, Jining No. 1 People’s Hospital, Jining, China; 4 Department of Rehabilitation Sciences, The Hong Kong Polytechnic University, Hong Kong, Hong Kong SAR, China; 5 Department of Global Public Health, Karolinska Institutet, Stockholm, Sweden; University of Thessaly Faculty of Medicine: Panepistemio Thessalias Tmema Iatrikes, GREECE

## Abstract

**Background:**

Appropriate sleep duration is essential for maintaining normal cognitive function, but evidence is scarce in rural elderly population. This study aims to determine the associations between sleep duration and cognitive function among older adults in rural China.

**Methods:**

This population-based cross-sectional study used data from the Confucius Hometown Aging Project in Shandong, China. Data on demographics, lifestyles, and chronic health conditions were collected through questionnaire surveys, clinical examinations, and laboratory tests. Sleep duration per day was classified into four groups (≤5 h, 6 h, 7 h, and ≥8 h). The Mini-Mental State Examination (MMSE) was used to assess the global and domain-specific cognitive function. Linear and logistic regressions were performed to determine the associations between sleep duration and cognitive function.

**Results:**

Compared with 6 h sleep per day, sleep ≤5 h per day was associated with a higher odd of cognitive impairment with odds ratio (95% confident interval) being 1.70 (1.05, 2.74), but the association was attenuated and insignificant after the adjustment of covariates. Compared to those with 6 h sleep, individuals reporting short (≤5 h) or long (≥ 8 h) sleep duration per day had a lower MMSE score, and the adjusted β coefficient (95% confidence interval) was −0.36 (−0.71, −0.02) for sleep ≤5 h and −0.68 (−1.06, −0.30) for sleep ≥8 h. The patterns were similar for cognitive subdomains in orientation, attention and calculation.

**Conclusions:**

Our study showed that abnormal sleep duration was associated with poor cognitive function in older adults in China. Specifically, longer or shorter sleep duration was associated with lower cognitive function.

## Introduction

As population ages, the number of people suffering from Alzheimer’s disease (AD) and dementia is increasing, which is expected to reach around 152 million by 2050 [1]. Dementia has an enormous impact on individuals, families, and society. The recent national cross-sectional study from China indicated that about 15.07 million individuals aged 60 years and over have dementia, including 9.83 million with AD, 3.92 million with vascular dementia and 1.32 million with other forms of dementia [2]. In China, the annual treatment cost of AD was 167.74 billion US dollars in 2015, which is expected to reach 1.8 trillion US dollars by 2050 [3]. As there is no cure treatment for AD, the identification of potentially modifiable risk factors of AD across life course is particularly important to inform preventive interventions. The 2020 report of the Lancet Commission suggested that sociodemographic characteristics (e.g., education), lifestyle factors (e.g., smoking, obesity, and physical inactivity), and disease status (e.g., hypertension, and traumatic brain injury) are the contributors to the incidence of dementia [4]. More research is warranted to explore other potential factors of dementia.

Sleep disturbances appear to increase with age as older people have increased sleep latency, increased early-morning awakening, increased sleep fragmentation, decreased sleep quality, and difficulty in maintaining sleep [5]. Importantly, recent studies have suggested that sleep disorders may be an early marker of AD pathology [6–10]. Meta-analysis of Positron Emission Tomography imaging studies revealed that the toxic proteins of AD, including amyloid β (Aβ) and tau, began to pathologically accumulate in the brain in 15–20 years before the onset of cognitive impairment [6]. Sleep also gradually changes during the preclinical stage of AD, and sleep disturbances play a critical role in tau generation and Aβ deposition [6,7]. In animal models, sleep loss directly leads to further Aβ deposition in both microglia and neurons [8]. In older adults without cognitive impairment, reduced nighttime sleep duration is associated with the increased risk of Aβ deposition which happens before cognitive impairment [9]. Human cerebrospinal fluid tau also increases over 50% during sleep deprivation, and chronic sleep deprivation increases tau pathology spreading in tau seeding and spreading model [10].

Population-based studies found that abnormal sleep duration, long sleep latency, and sleep fragmentation were related to accelerated cognitive decline [11–14]. The objective sleep monitoring found that a middle range of total sleep time may maintain longitudinal cognitive stability, while low and high values of total sleep time could lead to longitudinal cognitive decline [15]. Meanwhile, a meta-analysis of 22,187 people over 65 years of age who were at follow-up for up to 22.5 years found a U-shaped relationship between sleep duration and the risk of cognitive dysfunction, and individuals with sleep duration of 7–8 h per night were at the lowest risk of cognitive impairment, sleep duration shorter or longer than this was associated with a higher risk of cognitive impairment [16]. At present, there are inconsistencies in the optimal sleep duration for the elderly, and previous studies have suggested that 6–8 h of sleep per night is more appropriate for the stability of cognitive function [17,18].

While most of the studies on the relation of sleep parameters with cognitive function have been conducted among urban populations, and evidence is lacking in rural population. Studies have suggested that the prevalence of cognitive impairment is higher among rural than urban elderly populations [19–21]. Rural elderly people often have lower levels of education, mainly illiterate and primary school, resulting in poor cognitive reserve [19,20]. In addition, this population has a high prevalence of hypertension, diabetes, and hyperlipidemia, which are also important factors for cognitive impairment [19,20]. Later studies also found that the lack of effective exercise and effective cognitive protection behaviors such as adequate social activities may also lead to the increased risk of cognitive impairment among rural elderly people [21].

Thus, it is crucial to explore the modifiable factors associated with cognitive impairment in rural older adults. In this population-based cross-sectional study, we sought to determine the association between sleep duration and cognitive function in a rural elderly population in China.

## Methods

### Study population

The Confucius Hometown Aging Project (CHAP) is an ongoing project that provides long-term and continuous free health check-ups for the elderly over 60 years of age in Xing Long Zhuang community nearby Qufu, Shandong Province, China [22]. The project was launched in 2010 with the aim of systematically understanding the health behaviors of the elderly in rural areas, the process of ageing and functional deterioration, as well as the factors associated with these processes to achieve healthy aging. The CHAP was conducted by Jining First People’s Hospital and Jining Medical University in Shandong, China, in collaboration with the Aging Research Center at Karolinska Institutet-Stockholm University, Stockholm, Sweden.

This population-based study used data from the survey 2014–2016 of CHAP. Of all eligible subjects (*n* = 1521), 38 participants moved out of the area or had missing data on age, leaving 1483 participants for the current analysis. All participants underwent a series of medical examinations including a general physical examination, biochemical tests of blood and urine, as well as a standard self-administered questionnaire survey.

The CHAP study was approved by the ethics committee in the Jining No. 1 People’s Hospital, Shandong, China. Written informed consent was obtained from all participants, or in the case of cognitively impaired persons, from their next of kin. Research has been conducted in accordance with ethical principle expressed in the Declaration of Helsinki and its later amendments.

### Measurement of variables

#### Sleep duration.

Self-reported sleep duration was collected using the following question: How many hours a day on average do you sleep? Sleep duration per day was categorized into four groups: ≤ 5 h, 6 h, 7 h, and ≥8 h. Previous studies have suggested that 6 h of sleep was beneficial, and 6 h of sleep per day was used as the reference group [17,18,23].

#### Cognitive function.

The Mini-Mental State Examination (MMSE) was used to assess cognitive function [24]. The MMSE was collected from a 30-point questionnaire within six domains (10 points for orientation, 3 points for immediate recall, 5 points for attention and calculation, 3 points for delayed recall, 8 points for linguistic competence, and 1 point for special and drawing). A higher MMSE score indicated better cognitive function. The MMSE score <24 was considered as cognitive impairment [25,26].

#### Potential Confounders.

The potential confounders were selected based on literature. Information on potential confounders was collected including socio-demographics (e.g., age, sex, and education), health behavioral factors (e.g., current smoking and current alcohol drinking), body mass index, medical history (e.g., a physician’s diagnosis of hypertension, diabetes, hyperlipidemia, coronary heart disease, heart failure, chronic obstructive pulmonary disease [COPD], stroke, brain trauma, hypothyroidism, hearing problem, and cataract), and depressive symptoms.

Education level was defined based on the question of highest education, and education was categorized as illiteracy (no school), primary school (1–6 years of school), junior high school or above (≥7 years of school). Current smoking was defined as an answer yes to the question of current smoking. Currently drinking was defined as the frequency of current alcohol consumption of more than once per month. Body mass index (kg/m^2^) was measured as weight (in kg) divided by square height (in meter). The presence of depressive symptoms was defined as having a score ≥5 of the 15-item geriatric depression scale [27].

### Statistical analysis

Demographic characteristics of the study participants by sleep duration were compared using chi-square test for categorical variables and ANOVA for continuous variables. Linear regression models were used to estimate the associations of sleep duration with total MMSE score and the score for specific domains, and β coefficient and 95% confidence interval (CI) were used for the associations. Logistic regression models were conducted to assess the association between sleep duration and cognitive impairment with odds ratio (OR) and 95% CI being presented for the associations. Two models were performed: model 1 was unadjusted; model 2 was adjusted for the covariates selected based on a backward selection from the following variables: age, sex, education, smoking, alcohol drinking, body mass index, hypertension, diabetes, hyperlipidemia, coronary heart disease, heart failure, stroke, chronic obstructive pulmonary disease, hypothyroidism, brain trauma, hearing problem, cataract and depressive symptoms. In the case of multiple comparisons, the Bonferroni method was applied to correct the *P*-values. We considered a two-tailed *P* ≤ 0.05 to be statistically significant.

IBM SPSS Statistics for Windows (version 24.0) (Armonk, NY, USA: IBM Corp) was used for all statistical analyses.

## Results

Table 1 presented the demographic characteristics, lifestyles, and clinical factors of study participants. Of the 1483 participants, the mean age was 69.6 years and 66.4% were female. 30% of the participants had a sleep duration of 6 h per day. People with sleep duration of 6 h per day) were more likely to be younger on average, have better education and have higher MMSE scores. People with short sleep duration (≤5 h) were more likely to be female, drink less, have a history of coronary heart disease, hearing problem, and a higher prevalence of depressive symptoms. People with long sleep duration (≥8 h) had a lowest prevalence of hypothyroidism (*P* = 0.006). There were no significant differences in current smoking, hypertension, diabetes, hyperlipidemia, heart failure, COPD, stroke, brain trauma, cataract, and body mass index (all *P* > 0.05). The prevalence of cognitive impairment was 8.4% in total participants, and the group with sleep ≤5 h had the highest prevalence of cognitive impairment (*P* = 0.026).

**Table 1:** Demographic characteristics, lifestyles, and clinical factors of study participants.

Characteristics	Total	Sleep duration (hours)				
		≤5	6	7	≥8	P value
Number of participants	1483	384	437	280	382	
Age (year), mean (SD)	69.6(5.7)	70.5(5.6)	69.2(5.6)	69.0(5.4)	69.5(5.9)	0.001
Female sex, n(%)	984(66.4)	282(73.4)	281(64.3)	171(61.1)	250(65.4)	0.004
Educational levels, n(%)						0.004
Illiteracy	523(35.3)	166(43.2)	134(30.7)	102(36.4)	121(31.7)	
Primary school	625(42.1)	139(36.2)	193(44.2)	114(40.7)	179(46.9)	
Junior high school or above	335(22.6)	79(20.6)	110(25.2)	64(22.9)	82(21.5)	
Current smoking, n(%)	162(10.9)	33(8.6)	45(10.3)	44(15.7)	40(10.5)	0.076
Current drinking, n(%)	176(11.9)	25(6.5)	59(13.5)	37(13.2)	55(14.4)	0.002
Hypertension, n(%)	764(51.5)	191(49.7)	225(51.5)	151(53.9)	197(51.6)	0.768
Diabetes, n(%)	321(21.6)	93(24.2)	95(21.7)	48(17.1)	85(22.3)	0.177
Hyperlipidemia, n(%)	446(30.1)	135(35.2)	119(27.2)	83(29.6)	109(28.5)	0.077
Coronary heart disease, n(%)	259(17.5)	80(20.8)	80(18.3)	49(17.5)	50(13.1)	0.040
Heart failure, n(%)	91(6.1)	29(7.6)	21(4.8)	19(6.8)	22(5.8)	0.395
COPD, n(%)	198(13.4)	60(15.6)	59(13.5)	30(10.7)	49(13.6)	0.321
Stroke, n(%)	73(4.9)	23(6.0)	16(3.7)	12(4.3)	22(5.8)	0.357
Brain trauma, n(%)	38(2.6)	11(2.9)	8(1.8)	8(2.9)	11(2.9)	0.722
Hypothyroidism, n(%)	35(2.4)	12(3.1)	7(1.6)	13(4.6)	3(0.8)	0.006
Cataract, n(%)	288(19.4)	86(22.4)	84(19.2)	43(15.4)	75(19.6)	0.161
Hearing problem, n(%)	325(21.9)	106(27.6)	96(22.0)	51(18.2)	72(18.8)	0.020
BMI (kg/m^2^), mean(SD)	25.8(3.3)	25.7(3.2)	25.8(3.2)	25.6(3.6)	26.1(3.4)	0.271
Depressive symptoms, n(%)	390(26.3)	128(33.3)	123(28.1)	64(22.9)	75(19.7)	<0.001
MMSE scores, mean (SD)	27.9(2.9)	27.7(2.9)	28.3(2.4)	28.1(2.6)	27.7(3.5)	0.001
Cognitive impairment, n(%)	125(8.4)	44(11.5)	31(7.1)	15(5.4)	35(9.2)	0.026

Abbreviations: SD, standard deviation; COPD, chronic obstructive pulmonary disease; BMI, body mass index; MMSE, the mini-mental state examination

As shown in Table 2, compared to participants with a sleep time of 6 h/day, the β coefficient (95% CI) was −0.67 (−1.04, −0.30), −0.18 (−0.56, 0.20), and −0.70 (−1.11, −0.28) for sleep ≤5 h, 7 h, and ≥8 h, respectively. After accounting for potential confounders, the pattern remained almost the same in model 2.

**Table 2:** β coefficient (95% confidence interval) of Mini-Mental State Examination score associated with sleep duration.

Sleep duration (hours)	No. participants	Model 1	Model 2	Corrected P-value
≤5	384	−0.67(−1.04, −0.30)	−0.36(−0.71, −0.02)	0.014
6	437	0.00(Ref.)	0.00(Ref.)	–
7	280	−0.18(−0.56, 0.20)	−0.16(−0.52, 0.19)	0.123
≥8	382	−0.70(−1.11, −0.28)	−0.68(−1.06, −0.30)	<0.001

Model 1 was unadjusted;

Model 2 was a multivariate model.

Table 3 presented the association between sleep duration and cognitive impairment. In model 1, compared to participants with sleep of 6 h, participants who had sleep duration ≤5 h had an increased odd of cognitive impairment (OR = 1.70, 95% CI = 1.05, 2.74). After adjustment of confounders, the OR became attenuated and insignificant.

**Table 3:** Odds ratio (95% confidence interval) of cognitive impairment associated with sleep duration.

Sleep duration (hours)	No. participants	Model 1	Model 2	Corrected P-value
≤5	384	1.70(1.05, 2.74)	1.15(0.68, 1.97)	0.201
6	437	1.00(Ref.)	1.00(Ref.)	–
7	280	0.74(0.39, 1.40)	0.78(0.40, 1.55)	0.162
≥8	382	1.32(0.80, 2.19)	1.33(0.77, 2.32)	0.103

Model 1 was unadjusted;

Model 2 was a multivariate model.

Table 4 showed the association between sleep duration and each of the cognitive subdomains. The adjusted model (Model 2) showed that compared to 6 hours’ sleep, short (≤5 h) sleep duration per day was associated with a lower score in orientation, attention and calculation, and long (≥ 8h) sleep duration per day was associated with a lower score in orientation, attention and calculation, delayed recall, and linguistic competence.

**Table 4:** β-coefficient (95% confidence interval) of cognitive domain score associated with sleep duration.

Cognitive domain	Sleep duration (hours)	Model 1	Model 2	Corrected P-value
Orientation				
	≤5	−0.16(−0.27, −0.04)	−0.09(−0.21, 0.02)	0.039
	6	0.00(Ref.)	0.00(Ref.)	–
	7	−0.08(−0.21, 0.04)	−0.08(−0.20, 0.04)	0.068
	≥8	−0.15(−0.28, −0.03)	−0.13(−0.25, −0.009)	0.012
Immediate recall				
	≤5	0.002(−0.003, 0.01)	0.003(−0.002, 0.008)	0.071
	6	0.00(Ref.)	0.00(Ref.)	–
	7	−0.001(−0.01, 0.01)	−0.003(−0.01, 0.005)	0.175
	≥8	−0.01(−0.03, 0.01)	−0.01(−0.03, 0.01)	0.074
Attention and calculation				
	≤5	−0.36(−0.54, −0.19)	−0.25(−0.42, −0.08)	0.001
	6	0.00(Ref.)	0.00(Ref.)	–
	7	−0.04(−0.21, 0.12)	−0.04(−0.20, 0.12)	0.209
	≥8	−0.25(−0.42, −0.08)	−0.25(−0.40, −0.09)	<0.001
Delayed recall				
	≤5	−0.04(−0.11, 0.04)	−0.01(−0.09, 0.06)	0.247
	6	0.00(Ref.)	0.00(Ref.)	–
	7	−0.02(−0.10, 0.06)	−0.03(−0.11, 0.05)	0.160
	≥8	−0.09(−0.17, −0.01)	−0.09(−0.17, −0.01)	0.009
Linguistic competence				
	≤5	−0.10(−0.20, 0.004)	−0.05(−0.14, 0.05)	0.116
	6	0.00(Ref.)	0.00(Ref.)	–
	7	−0.04(−0.14, 0.07)	−0.03(−0.13, 0.07)	0.181
	≥8	−0.17(−0.30, −0.05)	−0.17(−0.29, −0.06)	0.001
Special and drawing				
	≤5	−0.05(−0.12, 0.02)	−0.02(−0.09, 0.05)	0.192
	6	0.00(Ref.)	0.00(Ref.)	–
	7	0.002(−0.07, 0.08)	0.01(−0.06, 0.08)	0.260
	≥8	−0.04(−0.11, 0.03)	−0.03(−0.10, 0.03)	0.113

Model 1 was unadjusted;

Model 2 was a multivariate model.

## Discussion

We found that sleep duration was associated with cognitive function. Compared with 6 h sleep per day, sleeping ≤5 h and ≥8 h per day was associated with lower scores of global cognition and specific domains such as orientation, attention and calculation.

Our population-based study showed that abnormal sleep duration was associated with poor cognitive function in rural older adults in China. Specifically, both short (≤5 h per day) and long (≥8 h per day) sleep durations were consistently associated with lower cognition scores. For example, a study used the data from two nationally representative aging cohorts in the United Kingdom and China found that individuals with sleep duration of ≤4 h and ≥10 h per night declined faster in global cognitive scores compared to those who slept 7 h per night [12]. Another study from the Chinese Longitudinal Healthy Longevity Survey (CLHLS) reported similar results that short (< 6 h) and long (> 8 h) sleep durations were positively associated with cognitive impairment compared to 7 h sleep duration per night [28]. The CLHLS study that sleeping >7 h per night was associated with cognitive impairment in both those with normal cognition and mild cognitive impairment at baseline during 4-year follow-up [28]. The Whitehall II study using a 25-year follow-up demonstrated that compared with a normal (7 h) sleep duration, short sleep duration (six hours or less) at age 50, 60, and 70 was associated with a 30% increased dementia risk [29]. These studies suggested that abnormal sleep duration is an independent risk factor for cognitive impairment.

The previous studies have found an inversed U-shaped relationship between sleep duration and cognitive function, sleep duration of 7 h was recommended as normal sleep duration [12,28,29]. These studies differ slightly from the optimal sleep duration of 6 h identified in our study. We found that compared with those who slept 6 h per day, those who slept shorter had a lower score in orientation, attention and calculation. However, several studies have also reached different conclusions regarding the effects of different sleep duration on cognitive subdomains. The National Health and Nutrition Examination Survey study also found that long nighttime weekday or workday sleep duration was associated with declined verbal memory, semantic fluency, working memory, and processing speed, and sleep duration of 10 h or more was associated with lower scores on immediate recall, delayed recall, animal fluency test, digital symbol substitution test, and greater odds of subjective cognitive problems [30]. The CLHLS study indicated that both long and short sleep durations were significantly associated with delayed recall, and long sleep duration (>9 h) was significantly associated with lower cognition in global and four cognitive domains: orientation, attention and calculation, immediate recall and visual construction [31]. The findings were partly consistent with ours that longer sleep time was associated with a lower score in orientation, attention and calculation, delayed recall, and linguistic competence. The reasons for the different results of these studies were mainly due to the variations in age and personnel composition of the study population, and different screening methods for cognitive function.

There are some potential mechanisms that may explain the association between sleep duration and cognitive function. Abnormal sleep cycles suggest changes in biological rhythms, and previous study showed that disturbed circadian function was associated with higher risk of preclinic AD pathology or dementia [32,33]. For example, insufficient nighttime sleep duration has been associated with higher burden in global Aβ, medial orbitofrontal Aβ and anterior cingulate Aβ before cognitive change [9]. Besides, middle-aged and older people with short and long sleep durations were reported to have more depressive symptoms, metabolic syndrome and cerebral small vessel disease, which have been found to be associated with an increased risk of cognitive impairment [34–36]. Moreover, in rural areas, the old population had the lowest educational level, higher prevalence rate of metabolic syndrome, higher proportion of alcohol consumption, smoking consumption and lower proportion of fish consumption [20,37]. The insufficient cognitive reserve of the rural elderly population, coupled with the high prevalence of metabolic syndrome and poor lifestyle habits, further aggravates vascular damage, leads to cerebral perfusion, and accelerates the occurrence and development of cognitive impairment. Additionally, lack of physical exercise and recreational activities at night may reduce cardiorespiratory function and neuroplasticity, lead to lower levels of brain-derived neurotrophic factor, and further prolong sleep time and damage cognitive function [19,38]. This study found that appropriate sleep cycles are particularly important for maintaining cognitive stability.

The study strengths included the population-based study design, big sample size, and comprehensive measurements of covariates. This study also had several limitations. Firstly, habitual sleep duration was measured based on self-reported questions, which could be biased. It may not accurately reflect the time from going to bed to falling asleep, or the time from waking up during the night to falling asleep again. Furthermore, there was a lack of information on the quality of sleep which could affect the associations between sleep duration and cognition. Secondly, this cross-sectional study is not sufficient to explain the causal relationship between sleep duration and cognitive impairment, and further cohort follow-up is needed to verify the results. Thirdly, only a single MMSE scale was used to measure cognitive function, which could not effectively identify early cognitive impairment due to its poor sensitivity. Fourth, although we adjusted for many covariates in the association, there might be still some residual confounding that could bias the associations. Fifth, our study participants were from rural area of China, and the study findings should be generalized with caution to the population with similar backgrounds.

## Conclusions

In conclusion, this population-based cross-sectional study suggested that shorter or longer sleep duration was associated with lower cognitive function in the rural elderly population. Given the significant impact of sleep duration on cognitive function, future studies are needed to explore the causality and mechanism of the association between abnormal sleep duration and cognitive decline.
